# Fixed-Bed Adsorption of As and Pb Using Carbon Nanotubes, Activated Carbon and Zeolite: Solids Characterization and Kinetic Analysis

**DOI:** 10.3390/nano16160975

**Published:** 2026-08-08

**Authors:** Miriam Estrada, Rubén H. Olcay, Iván Alejandro Reyes, Francisco Patiño, Hernán Islas, Mizraim U. Flores, J. Eliecer Méndez, Gildardo Godínez, Sayra Ordoñez

**Affiliations:** 1 Institute of Metallurgy, Autonomous University of San Luis Potosí, San Luis Potosí 78210, Mexico; alejandro.reyes@uaslp.mx; 2 Industrial Electromechanics Area, Technological University of Tulancingo, Tulancingo 43642, Mexico; mflores@utectulancingo.edu.mx (M.U.F.); eliecer@utectulancingo.edu.mx (J.E.M.); 3 Department of Metallurgical Engineering, Faculty of Engineering and Architecture, Arturo Prat University, Arturo Prat Avenue 2120, Iquique 1110939, Chile; rolcay@unap.cl; 4 SECIHTI Secretariat of Science, Humanities, Technology, and Innovation, Mexico City 03940, Mexico; 5 Energy Engineering, Metropolitan Polytechnic University of Hidalgo, Tolcayuca 43860, Mexico; franciscopatinocardona@gmail.com (F.P.); hernan.islas@hidalgo.gob.mx (H.I.); 6 Academic Area of Computing and Electronics, Autonomous University of Hidalgo State, Mineral de la Reforma 42183, Mexico; gildardo_godinez@uaeh.edu.mx; 7 University Center for Computer Engineering, Autonomous University of Mexico State, Zumpango 55600, Mexico

**Keywords:** heavy metals, carbon nanotubes, zeolite, activated carbon, adsorption kinetics, fixed-bed

## Abstract

This study presents a fixed-bed column packed with zeolite, activated carbon, and carbon nanotubes synthesized by chemical vapor deposition, which were used to adsorb heavy metals (As and Pb) from water. Water containing 50 mg/L of the target heavy metal was filtered through the column. The materials were characterized before and after their use in the column by X-ray diffraction (XRD) and scanning electron microscopy coupled with energy-dispersive X-ray spectroscopy (SEM-EDS). The analyses confirmed the structural stability and functional efficiency of each adsorbent material. A removal efficiency of 100% was achieved within the first 15 min for both metals, decreasing to 45% for As and 95% for Pb. The experimental data were evaluated using the Thomas, Yoon–Nelson, and Adams–Bohart models. For arsenic, an R^2^ value of 0.934 was obtained, whereas better fits were observed for Pb, with all three models achieving R^2^ values greater than 0.98. Dynamic adsorption analysis indicated that the mass and height of the fixed bed are suitable for the operating concentration. The column arrangement proved to be more efficient for Pb retention than for As retention.

## 1. Introduction

Water, which is essential for the health, well-being, and sustainability of humans and ecosystems, is the most important public good. However, inadequate water supply, as well as the high costs of purification, have led to the use of untreated groundwater and rainwater for consumption and domestic applications. Civilization expansion, population growth, industry, agriculture, and poor wastewater disposal have worsened water quality [1]. The mining industry releases many pollutants, such as heavy metals (arsenic, chromium, cadmium, mercury, lead, and zinc, among others), which end up being deposited in surface or groundwater bodies [2]. Heavy metals represent a threat to water quality and aquatic ecosystems, and they are also a risk to human health because they are difficult to remove, cannot be degraded, and are persistent [3]. Both arsenic (As) and lead (Pb) are considered toxic at high concentrations, and their widespread use can cause health problems, impacting the nervous system, kidneys, and blood circulation, particularly during early childhood [4]. Therefore, the WHO [5] has established a maximum permissible exposure limit of 0.01 mg/L for both As and Pb. Consequently, removing toxic heavy metals from water is required for health and environmental sustainability [6]. Many technologies remove or degrade water contaminants. These include precipitation [7], photocatalytic degradation for organic contaminants [8], electrolysis [9], biological separation, adsorption [10], and oxidation [11]. However, these methods have drawbacks, such as chemical use, high energy consumption, and high costs. Engineering skills and infrastructure are needed for mitigation [12,13], thus creating a need for effective, eco-friendly methods. Adsorption is widely used for its simplicity, efficiency, low environmental impact, ease of operation, and the ability to regenerate adsorbents without performance loss [14,15,16,17].

Many adsorbents, both native and modified, remove heavy metals. Activated carbons (ACs), zeolites, and advanced adsorbents have been tested for this purpose [18]. ACs are made from non-graphitizable materials with structural disorder, giving them porosity (micropores < 2 nm to mesopores 2–50 nm), surface chemistry, free valences, hydrophobicity, and surface groups. These traits make them useful for adsorption [19]. Heavy metals bind to ACs by physical adsorption, electrostatic attraction, ion exchange, reduction, complexation, or precipitation [20]. Chemical and physical modifications increase ACs’ affinity for various metals. Zeolites, with a three-dimensional skeleton of SiO_4_ and AlO_4_ units and weakly bound water/cations, have a large surface area and ion-exchange properties [21,22]. They are used in water softening and heavy metal removal. Zeolites trap heavy metals on their surface and within pores. Carbon nanotubes (CNTs) also show promise as adsorbents for many metal ions [23,24,25]. Mathematical models help evaluate the efficiency of heavy metal adsorption and interpret how metal ions interact with adsorbents, depending on the adsorbent structure and solute properties [26,27].

This study presents the adsorption capacity of carbon nanotubes (CNTs), activated carbon, and zeolite for the removal of arsenic (As) and lead (Pb) from water in a continuous fixed-bed column. The materials were characterized (XRD and SEM/EDS) before and after use. The system was operated without changes in pH or temperature. The dynamic adsorption behavior of the fixed-bed column was evaluated, and the data were analyzed using the Thomas, Yoon–Nelson, and Adams–Bohart models to develop a scalable, continuous fixed-bed adsorbent column that does not require recovery of suspended particles by filtration, sedimentation, or other methods after use.

## 2. Materials and Methods

### 2.1. Chemical Vapor Deposition Synthesis of Carbon Nanotubes

NTC synthesis was performed by chemical vapor deposition (CVD) using a mixture of ferrocene, ethanol, and toluene (5%, 25%, and 92.5%, respectively). Synthesis was performed at 850 °C for 20 min using 20 mL of source solution and an argon flow rate of 1380 mL/min. The argon flow was maintained for 15 min, and 1 mL of solution was injected into the ultrasonic nebulization chamber every minute. Once the synthesis time had elapsed, the argon flow was maintained for 30 min until the furnace reached room temperature [28].

### 2.2. Characterization of Materials

The structural and morphological features of carbon nanotubes, activated carbon, and zeolite were analyzed before and after using X-ray diffraction (XRD) and a scanning electron microscope (SEM). For XRD, a Bruker D8 powder diffractometer (Billerica, MA, USA) was used with Ni-filtered radiation from a Cu anode, kα1 λ = 1.5406 Å, 40 kV and 35 mA, a recorded angular range of 2θ of 5–90°, a step size of 0.01°, and a duration of 3 s per step. Diffract Eva software v7.0. and the ICDD-PDF-2 Release 2022 database were used for analysis. SEM analysis used a JEOL JSM-670 IF FESEM-EDX (Tokyo, Japan).

### 2.3. Column Packing

For the packed column, a polymer test tube was used, from which the base was removed, yielding a tube 3.5 cm in diameter and 30 cm long. To retain the nanotubes, a 0.45 µm PTFE membrane filter was assembled at the bottom as a sieve. This filter was 3.5 cm in diameter, 1 cm thick, and included a 1 cm long, 0.1 cm diameter tube that allowed water to drain. The column was packed with 0.1 g of nanotubes, 5 g of ultrafine activated carbon, and 5 g of zeolite which was pulverized to a size of 53 to 73 µm. A mortar and pestle was used to reduce the particle size of the activated carbon and zeolite. A 450 nm cellulose nitrate filter paper was placed between each material to separate the phases. The bed height was packed at 5 cm.

### 2.4. Atomic Absorption Spectroscopy

The solutions filtered through the column were analyzed for arsenic (As) and lead (Pb) using a PerkinElmer AAnalist 200 atomic absorption spectrometer (PerkinElmer, Inc., Shelton, CT, USA) with a hollow cathode lamp for lead analysis and an electrodeless discharge lamp for arsenic analysis. A 1000 mg/L standard solution of As and Pb was used to generate the calibration curve with serial concentrations (10, 40, and 80 mg/L).

### 2.5. Adsorption Efficiency

To measure the adsorption capacity of As and Pb ions, the properties of the packed bed system were evaluated. One hundred milliliters of solution was prepared from a 1000 mg/L stock solution of arsenic (pH = 2.74) and lead (pH = 2.16) at a concentration of 50 mg/L. An amount of 100 mL of solution was poured into the corresponding packed column, allowing it to flow through the filter media by gravity and collect at the bottom. The total filtration time was 3 h, and a sample was collected every 15 min. The flow rate of the solutions was calculated by measuring the volume of As and Pb obtained. Each filtered sample was analyzed by atomic absorption spectroscopy (AAS) to determine the remaining metal ions in the effluent. The adsorption capacity (q_t_) over time and the heavy metal removal capacity (%R) of the packed column were estimated using Equations (1) and (2):

(1) $$q_{t}=\;\frac{\left(C_{o}\;-\;C_{t}\right)V}{m}$$

(2) $$\%\;\mathrm{Removed}=\frac{\;\left(C_{o}\;-\;C_{t}\right)}{C_{o}}\;\times \;100$$

where q_t_ is the amount of metal ion adsorbed by the adsorbent materials of the column (mg/g), C_o_ is the initial concentration of the metal ion (mg/L), C_t_ is the final concentration of the metal ion after a certain time (mg/L), V is the initial volume (L) and m is the amount of adsorbent (g) [29].

### 2.6. Dynamic Adsorption Analysis

The adsorption efficiency of the continuous fixed-bed column was evaluated using the breakthrough curve, which includes the total volume of treated effluent, the breakthrough capacity, the removal efficiency, and the length of the mass-transfer bed. The time it takes for the effluent concentration to reach 10% of the influent concentration is known as the breakthrough time (t_b_), while the time it takes for the effluent concentration to reach 90% of the influent concentration is considered the depletion time (t_e_). The breakthrough curve is described by C_eff_/$C_{o}$, where C_eff_ is the effluent concentration and $C_{o}$ is the influent concentration. To determine the concentration of the adsorbed ions, a graph was generated from the concentration of the adsorbed metal (C_ad_ = initial concentration ($C_{o}$) − outlet concentration (C_eff_)) or normalized concentration, which is defined as the ratio between the concentration of the metal in the effluent and the concentration of the influent (C_eff_/C_O_) as a function of time or effluent volume (V_eff_), as indicated in Equation (3) [30]:

(3) $$V_{\mathrm{eff}}\left(\mathrm{mL}\right)\;=\;{\mathrm{Qt}}_{\mathrm{total}}$$

where Q and t_total_ represent the flow rate (mL/min) and the total flow time (min). The concentration of adsorbed ions (q_total_) is obtained from the graph of adsorbed concentration (C_ad_) vs. time (t). The area (A) under the integrated graph is substituted into Equation (4) to obtain q_total_.

(4) $$q_{\mathrm{total}}\left(\mathrm{mg}\right)=\frac{\mathrm{QA}}{1000}=\frac{Q}{1000}=\int_{t=0}^{t_{\mathrm{total}}}C_{\mathrm{ad}}\mathrm{dt}$$

The total mass of the metal arranged in the column (m_total_) is obtained from Equation (5).

(5) $$m_{\mathrm{total}}\left(\mathrm{mg}\right)=\frac{C_{o}Qt_{\mathrm{total}}}{1000}$$

To evaluate the column performance, the total percentage of ion removal is calculated from the total ions adsorbed on the column relative to the initial amount, as shown in Equation (6).

(6) $${\;\mathrm{Removed}}_{\mathrm{total}}\left(\%\right)=\frac{q_{\mathrm{total}}}{m_{\mathrm{total}}}\cdot 100$$

#### Modeling Studies on Fixed-Bed Column

Thomas, Yoon–Nelson, and Adams–Bohart models, represented by Equations (7)–(9), were employed to explain the adsorption mechanism and determine the appropriate model.

(7) $$\ln\left(\frac{C_{o}}{C_{t}}-1\right)=\frac{k_{\mathrm{Th}}q_{e}m}{Q}-k_{\mathrm{Th}}C_{o}t$$

(8) $$\ln\left(\frac{C_{t}}{C_{o}-C_{t}}\right)=k_{\mathrm{YN}}t-k_{\mathrm{YN}}t\tau_{50}$$

(9) $$\ln\left(\frac{C_{t}}{C_{o}}\right)=k_{\mathrm{AB}}C_{o}t-k_{\mathrm{AB}}N_{0}\frac{z}{U}$$

where $C_{o}$ represents the influent adsorbate concentration (mg/L), $C_{t}$ is the effluent concentration at time (mg/L), and t is the contact time. For the Thomas model, the rate constant is denoated as k*_TH_* (mL·mg^−1^·min^−1^), $q_{e}$ is the maximum adsorption capacity of the adsorbent (mg/g), *m* is the mass of adsorbent packed in the column (g), and *Q* is the volumetric flow rate. For the Yoon–Nelson model, $k_{\mathrm{YN}}$ is the rate constant (min^−1^) and *τ* is the time required for the effluent concentration to reach 50% of the influent concentration [31,32,33]. The Adams–Bohart model is used to describe the initial part of the breakthrough curve; the kinetic constant is $k_{\mathrm{AB}}$, $N_{0}$ is the maximum volumetric adsorption capacity of the adsorbent (mg/L), *z* represents the bed height (cm), and U is the superficial velocity (cm/min).

## 3. Results

### 3.1. Characterization of Adsorbent Materials

#### 3.1.1. X-Ray Diffraction Analysis (XRD) and Scanning Electron Microscopy Analysis (SEM) Before Packaging

**Figure 1:**
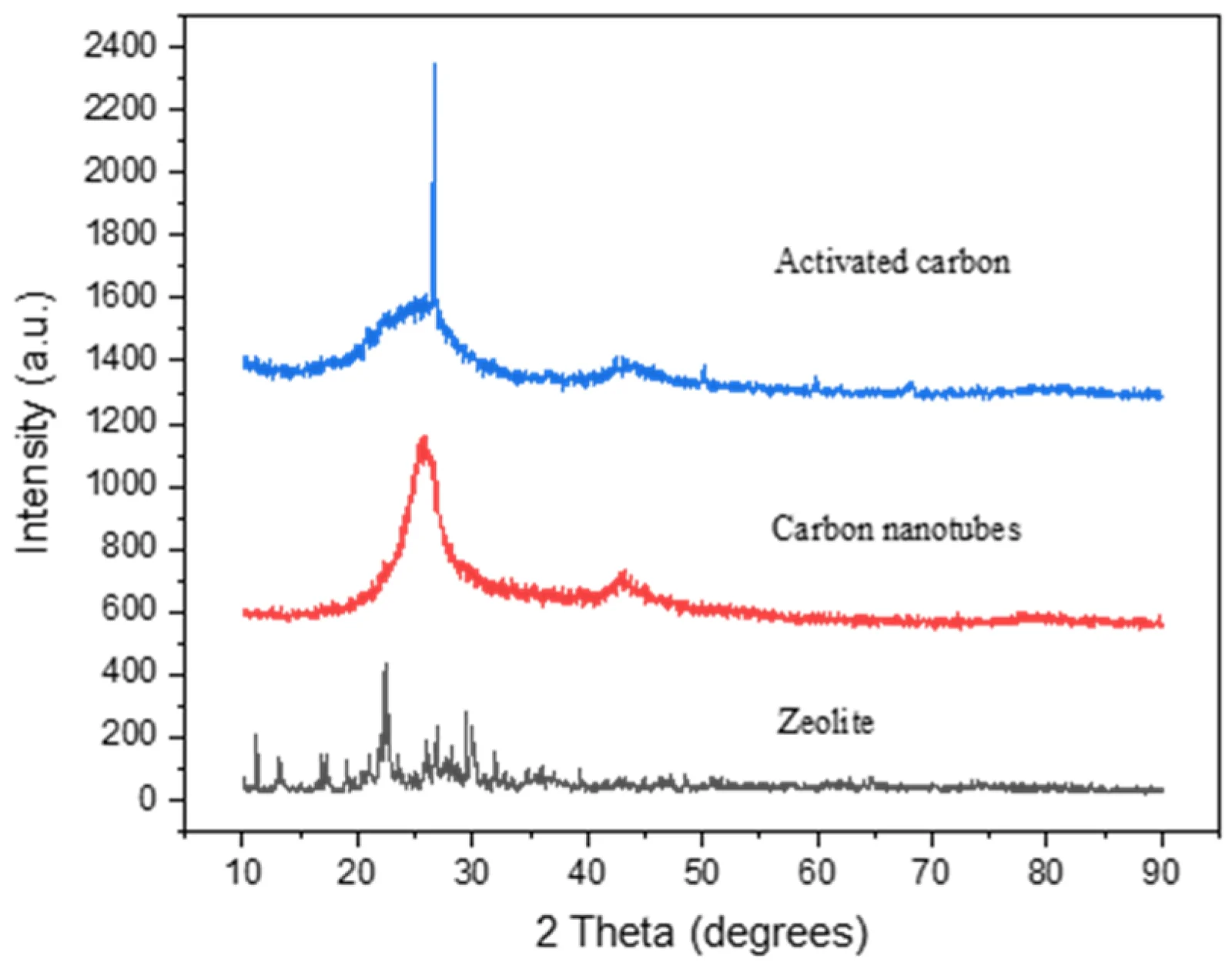
X-ray diffraction patterns of each material used to pack the filtration column.

Figure 1 shows the three X-ray diffraction patterns used to identify the phases present in each of these materials. The XRD pattern of the zeolite shows clinoptilolite (ICDD-PDF: 96-900-9579) as the major phase. Clinoptilolite is a characteristic mineral with a porous, three-dimensional network structure of SiO_4_ and AlO_4_ tetrahedra. The combination of its physicochemical properties, including large specific surface area, uniform pore size, ion exchange capacity, and thermal and chemical stability, makes it ideal for environmental applications [34], such as water and air purification, agricultural soil improvement, animal detoxification, and human supplements (micronized) for removing heavy metals and toxins, acting as a magnet for contaminants due to its negative charge. The diffraction pattern shows the typical reflections of activated carbon, which are consistent with its amorphous structure. Furthermore, two large, broad peaks are observed near 2θ = 26° and 2θ = 43°, attributed to reflections from the (002) and (100) planes (ICDD-PDF: 96-110-0004). The 24° peak in the activated carbon X-ray diffraction (XRD) pattern indicates an amorphous or turbostratic carbon structure, representing X-ray reflection from the (002) crystal plane. This is a broadened and shifted version of the sharp 26.5° peak of graphite, caused by the structural disorder and high porosity developed during the activation process. In perfect graphite, the graphene layers are perfectly aligned. In activated carbon, these layers are randomly displaced and rotated relative to each other, exhibiting significant layer disorder [35,36,37,38,39]. Activated carbon exhibits a narrow and prolonged peak, which originates from the presence of crystalline material. The amount of crystalline phases is very low and is attributed to the sample substrate/support, impurities caused by sample processing steps, or impurities present in the base material.

**Figure 2:**
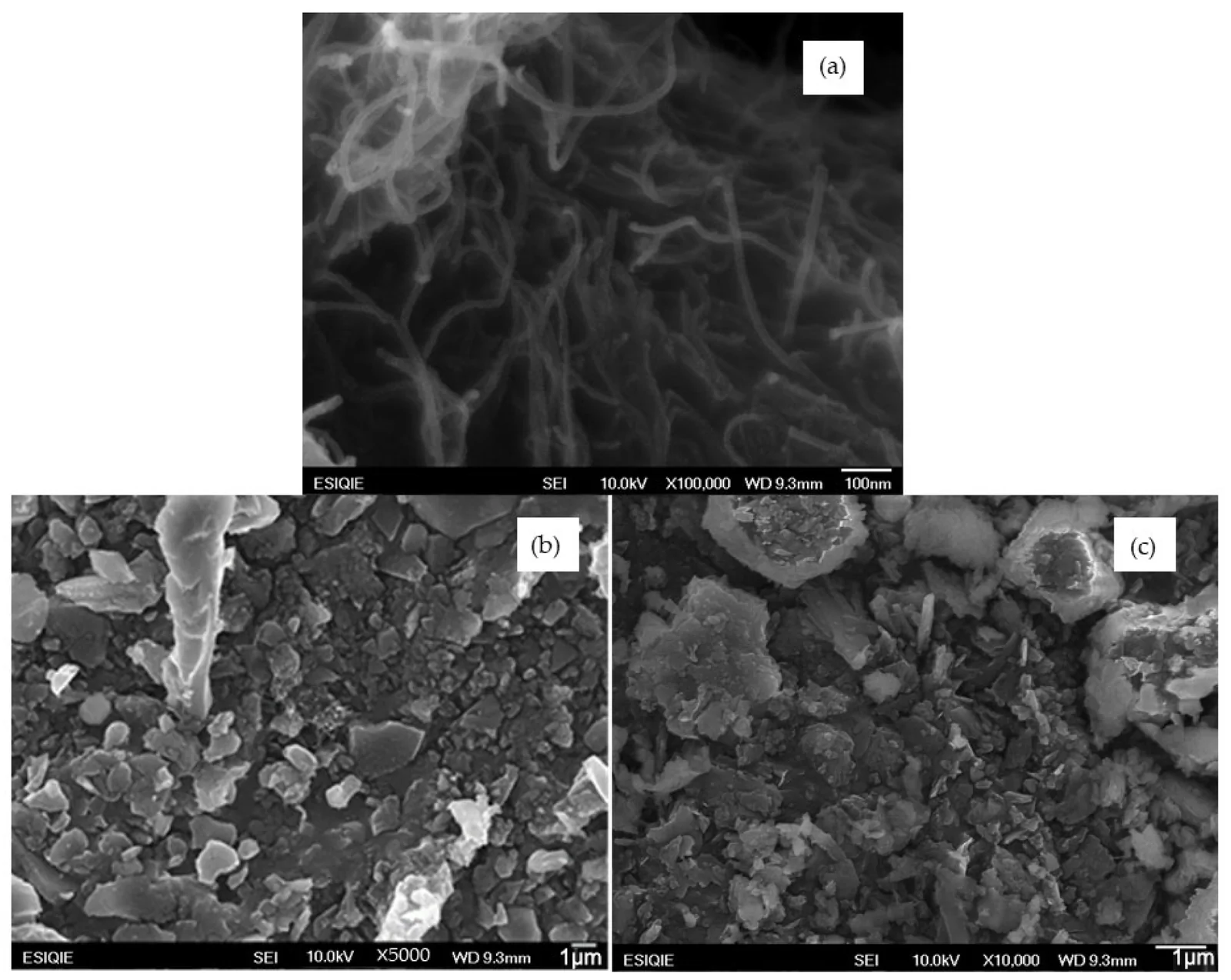
SEM images of (**a**) carbon nanotubes at 100,000× magnification, (**b**) activated carbon particles with different sizes, and (**c**) zeolite particles with different sizes.

Figure 2 shows SEM images of each of the materials used to pack the column. Figure 2a shows carbon nanotubes approximately 5 nm in diameter and several microns long. The carbon nanotubes have a porous texture, which is visible and homogeneous throughout the nanotubes. Figure 2b shows activated carbon with varying particle sizes, as particle size is not limited. Polygonal cross-sections are visible on the different faces of the carbon, and it also exhibits a porous texture. Figure 2c shows zeolite with different particle sizes, also demonstrating polygonal cross-sections and no porosity in the particles.

#### 3.1.2. X-Ray Diffraction Analysis (XRD) and Scanning Electron Microscopy Analysis (SEM) for NTC After Passing Through the Column

**Figure 3:**
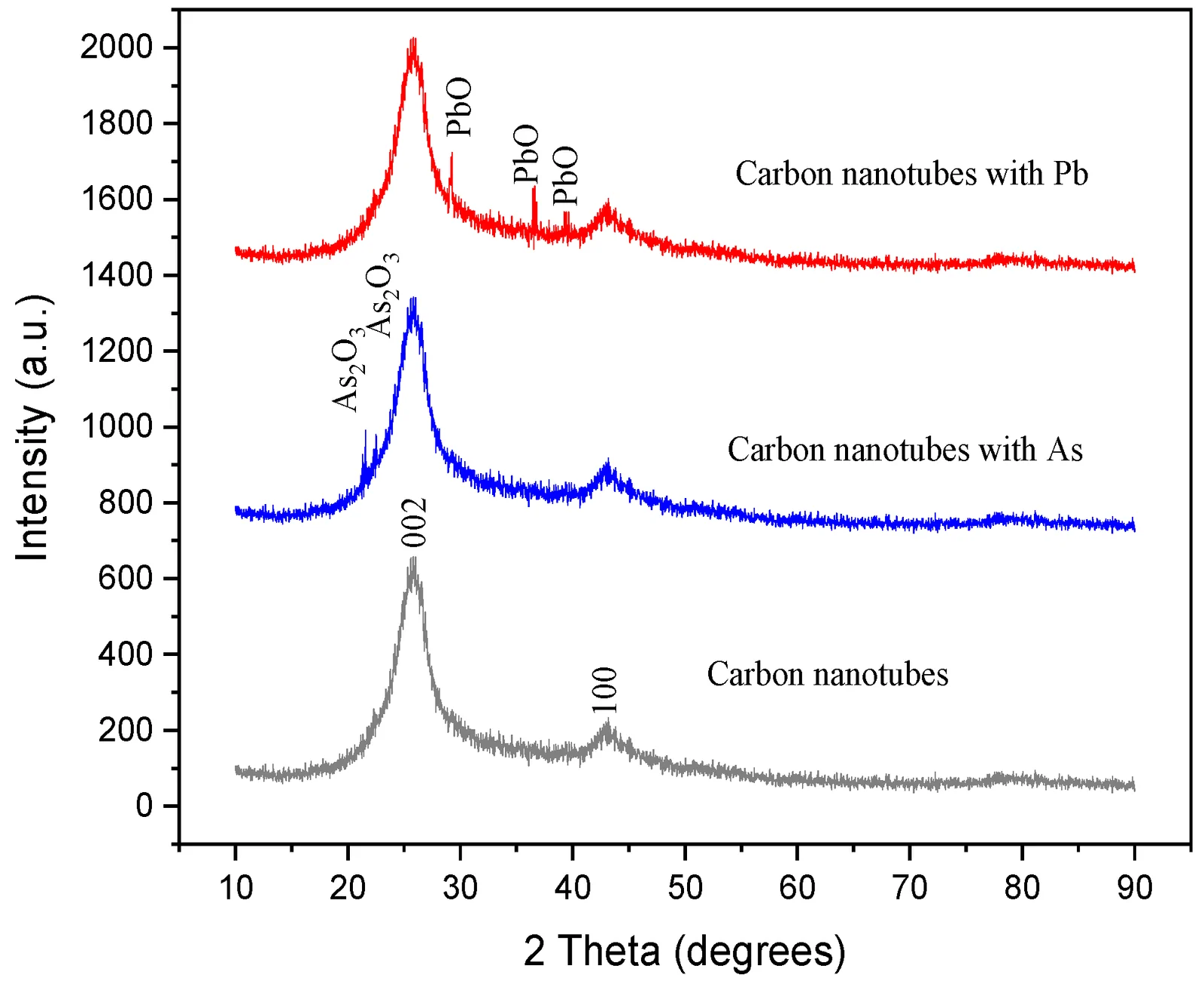
X-ray diffraction patterns of carbon nanotubes after use in the filtration column for the retention of arsenic and lead.

**Figure 4:**
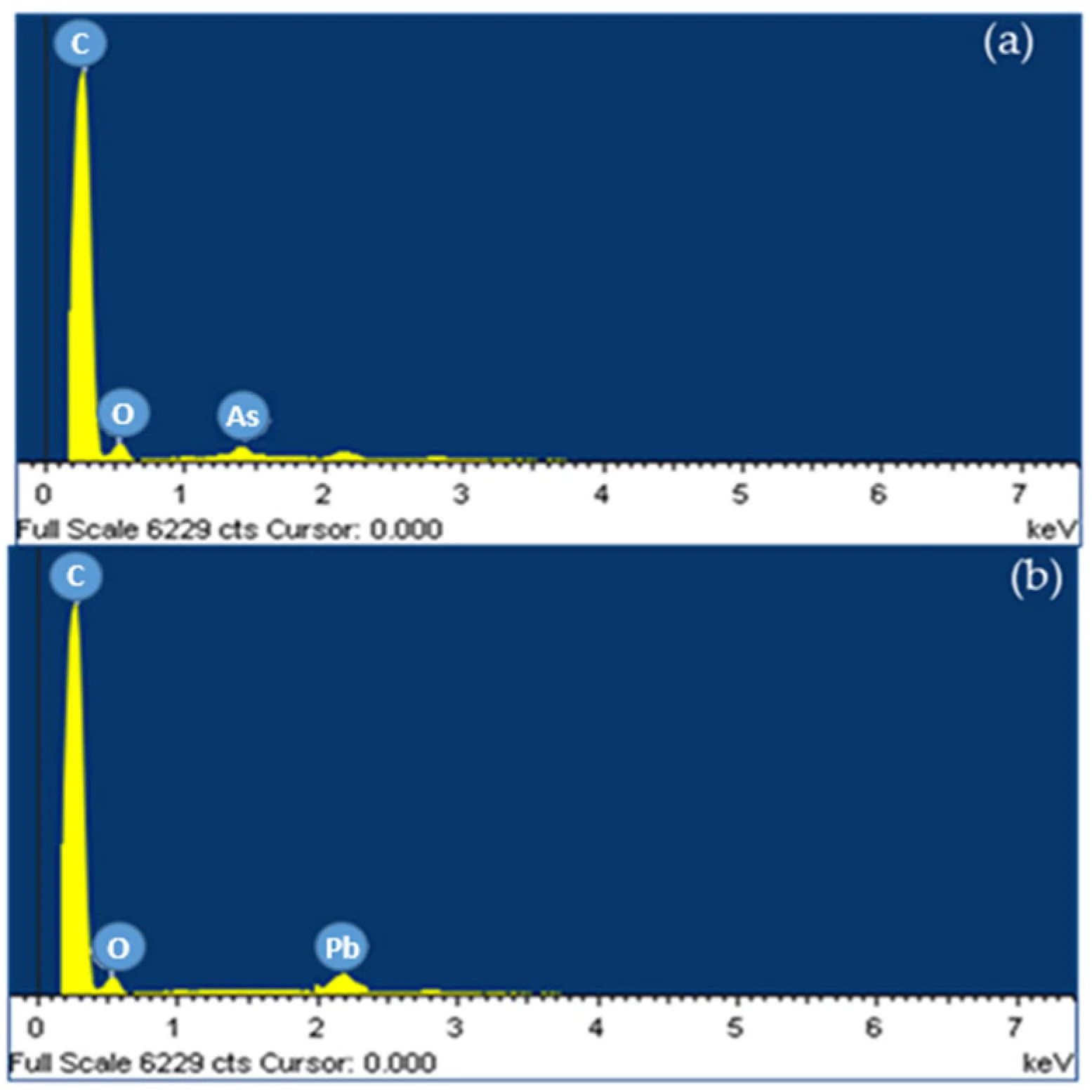
EDS spectra of carbon nanotubes showing the retention of both (**a**) arsenic and (**b**) lead in their structure.

The X-ray diffraction pattern of the carbon nanotubes (Figure 3) shows a new phase in those used for both arsenic and lead adsorption. The new phase present in the carbon nanotubes used to adsorb arsenic is arsenate (PDF: 96-450-6960), which shows signals around 20 degrees, similar to what has been reported by other authors [40]. The carbon nanotubes used to adsorb lead exhibit a new crystallographic phase of lead oxide (PDF: 96-900-7711) that forms at the interface between the column and the nanotubes. The crystallographic planes of lead oxide are located in the 30–40 degree region. EDS analysis shows that carbon nanotubes, in addition to containing carbon and oxygen, also contain arsenic (Figure 4a) and lead (Figure 4b), confirming that they retain these toxic elements.

#### 3.1.3. X-Ray Diffraction Analysis (XRD) and Scanning Electron Microscopy Analysis (SEM) for CA After Passing Through the Column

**Figure 5:**
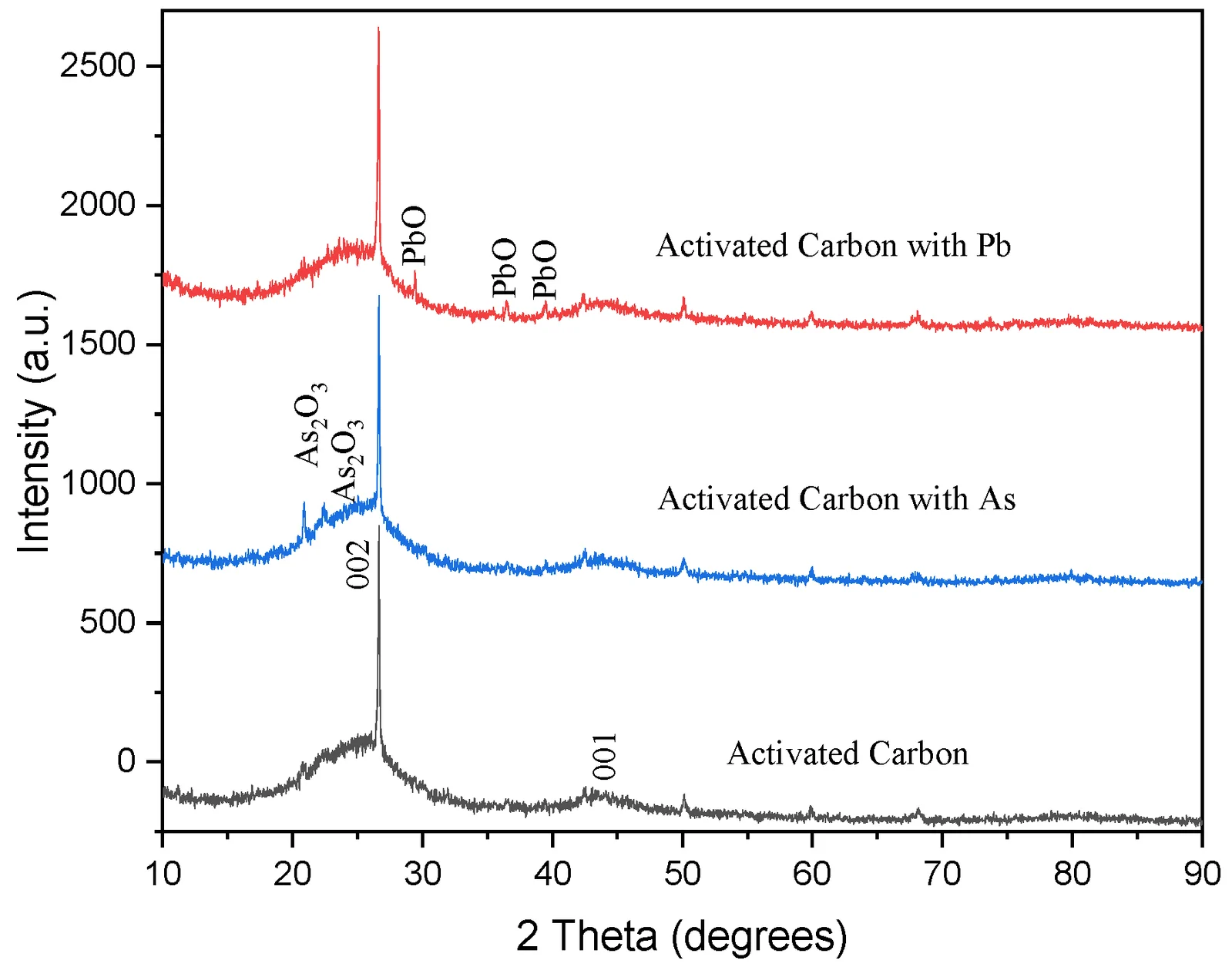
X-ray diffraction pattern of activated carbon after being used in the filtration column to retain arsenic and lead.

The same XRD analysis was performed on activated carbon, comparing its pattern before and after use in the column. This analysis also revealed the same crystallographic planes as those of the carbon nanotubes, indicating the presence of new phases containing the toxic elements targeted for retention. The formation of crystalline phases occurs due to the interaction of the toxic elements with the activated carbon surface and the water in which they are dissolved. The peaks in the X-ray diffraction pattern are less intense than those observed in the carbon nanotubes, suggesting that the nanotubes have a higher retention efficiency. The small peaks that appear in a 50–70 degree range could be attributed to contaminants from the preparation of activated carbon or to contaminants from the base material. Arsenic generally does not bond to the carbon of the nanotube to form a new and permanent chemical compound (such as a carbon–arsenic covalent bond in its structure). Instead, arsenic, in combination with oxygen from water and the environment, forms As_2_O_3_, a surface compound observed by X-ray diffraction in Figure 5. The lead ion in aqueous solution (Pb^2+^) binds to the surface of the nanomaterial, forming intermolecular bonds, ion-exchange bonds, or surface organometallic complexes (chemically represented as O-Pb).

**Figure 6:**
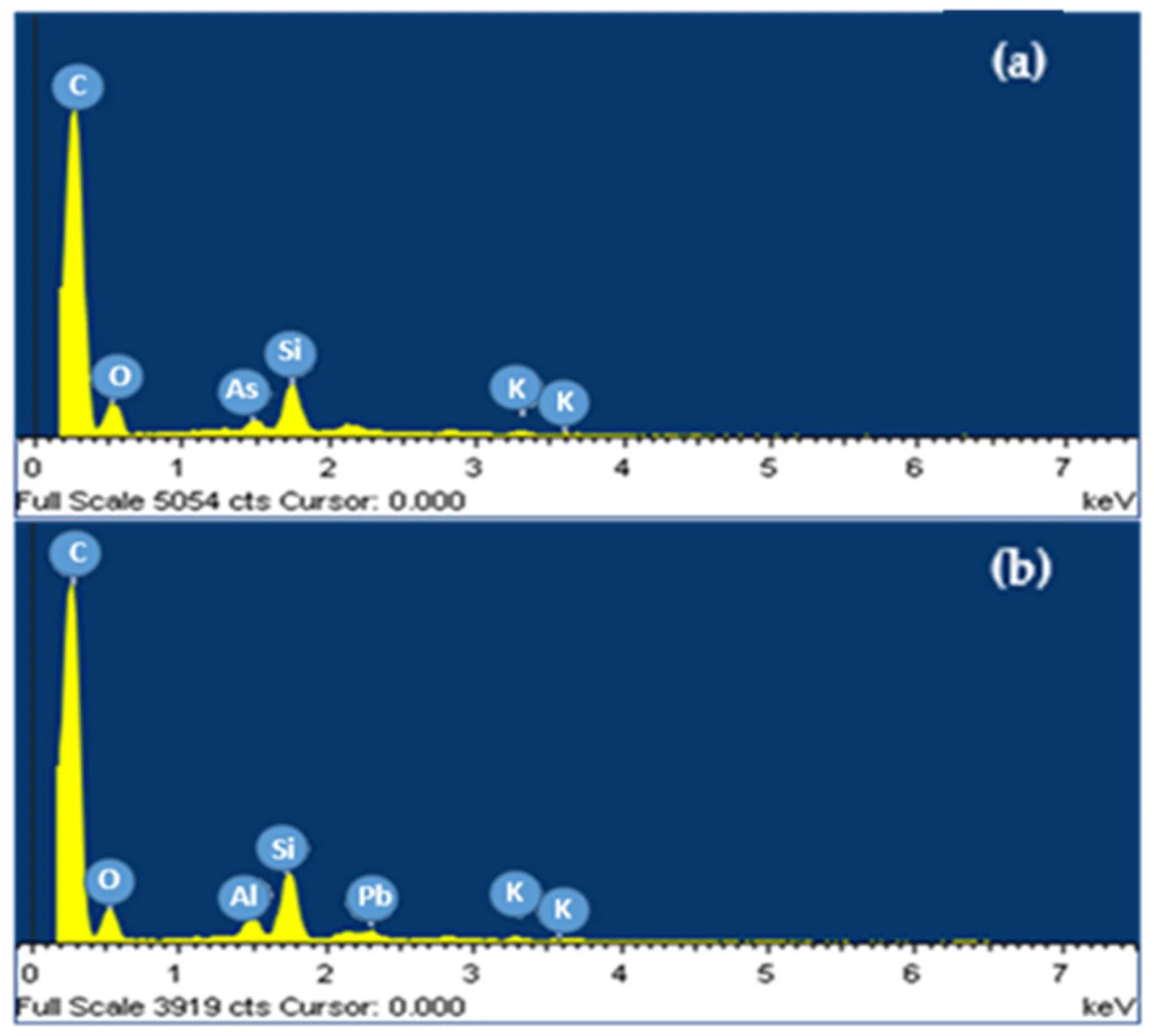
EDS spectra of activated carbon showing (**a**) arsenic and (**b**) lead retained in its structure.

The EDS spectra show the presence of arsenic (As) and lead (Pb) in the activated carbon. The signals are weak due to their poor retention capacity; however, they are functional for retaining these toxic elements. Figure 6 shows the characteristic elements of activated carbon, as well as the toxic elements. However, aluminum (Al), silicon (Si), and potassium (K) are also present due to the combination of some zeolite with the activated carbon. Carbon nanotubes retain arsenic through adsorption, a process where contaminant molecules become trapped on the surface of the nanomaterial. Their effectiveness stems from their enormous surface area, hollow structure, and the possibility of chemically modifying them to increase their attraction for arsenic ions. The massive surface area of carbon nanotubes and their cylindrical structure at the nanoscale give them an extremely large surface-area-to-volume ratio, providing millions of microscopic “binding sites” to capture heavy metals, specifically arsenic and lead. The available nanotubes are approximately 5 nanometers in diameter and several micrometers long, resulting in a large surface area.

#### 3.1.4. X-Ray Diffraction Analysis (XRD) and Scanning Electron Microscopy Analysis (SEM) for Zeolite After Passing Through the Column

**Figure 7:**
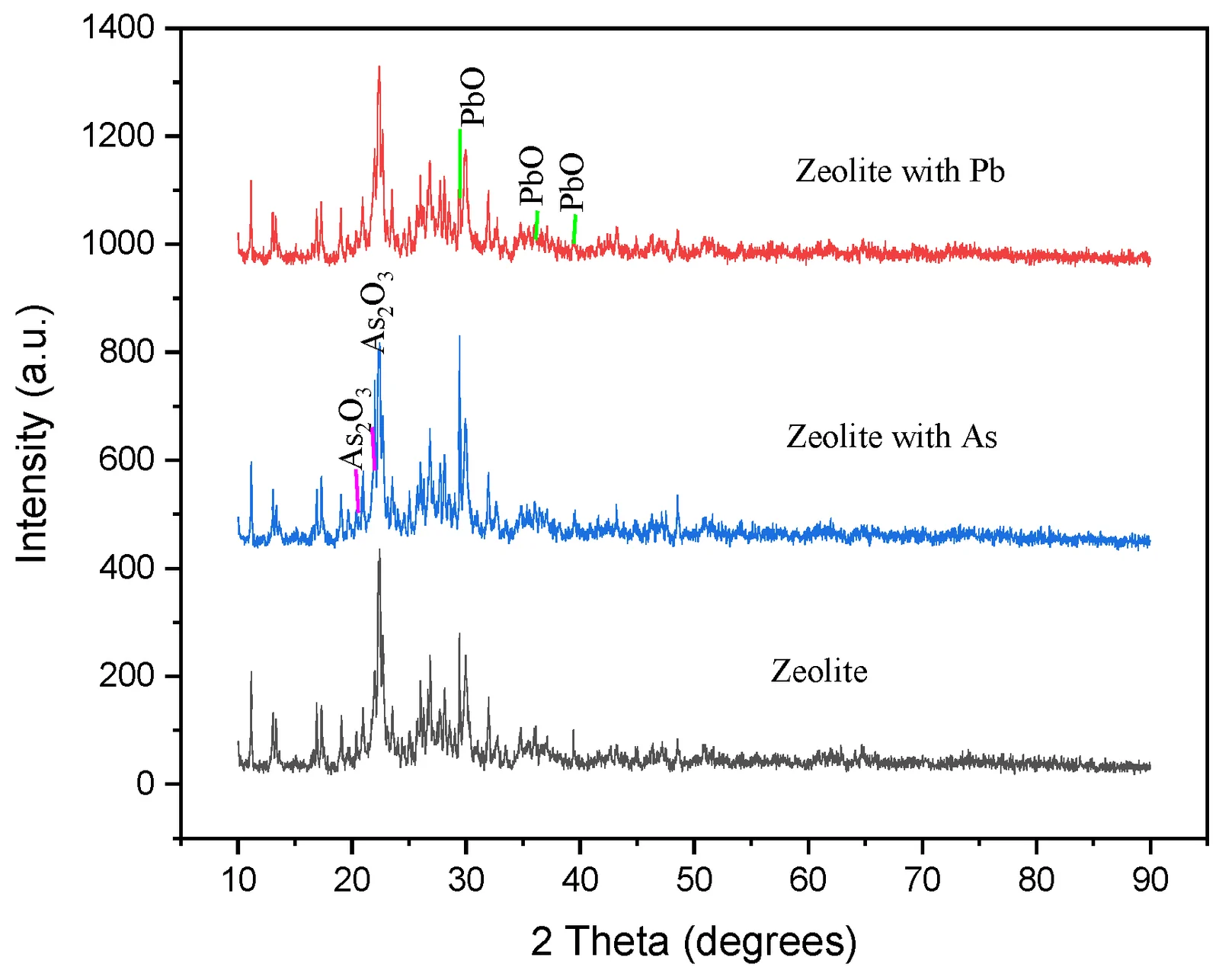
X-ray diffraction pattern of zeolite after being used in the filtration column to retain arsenic and lead.

**Figure 8:**
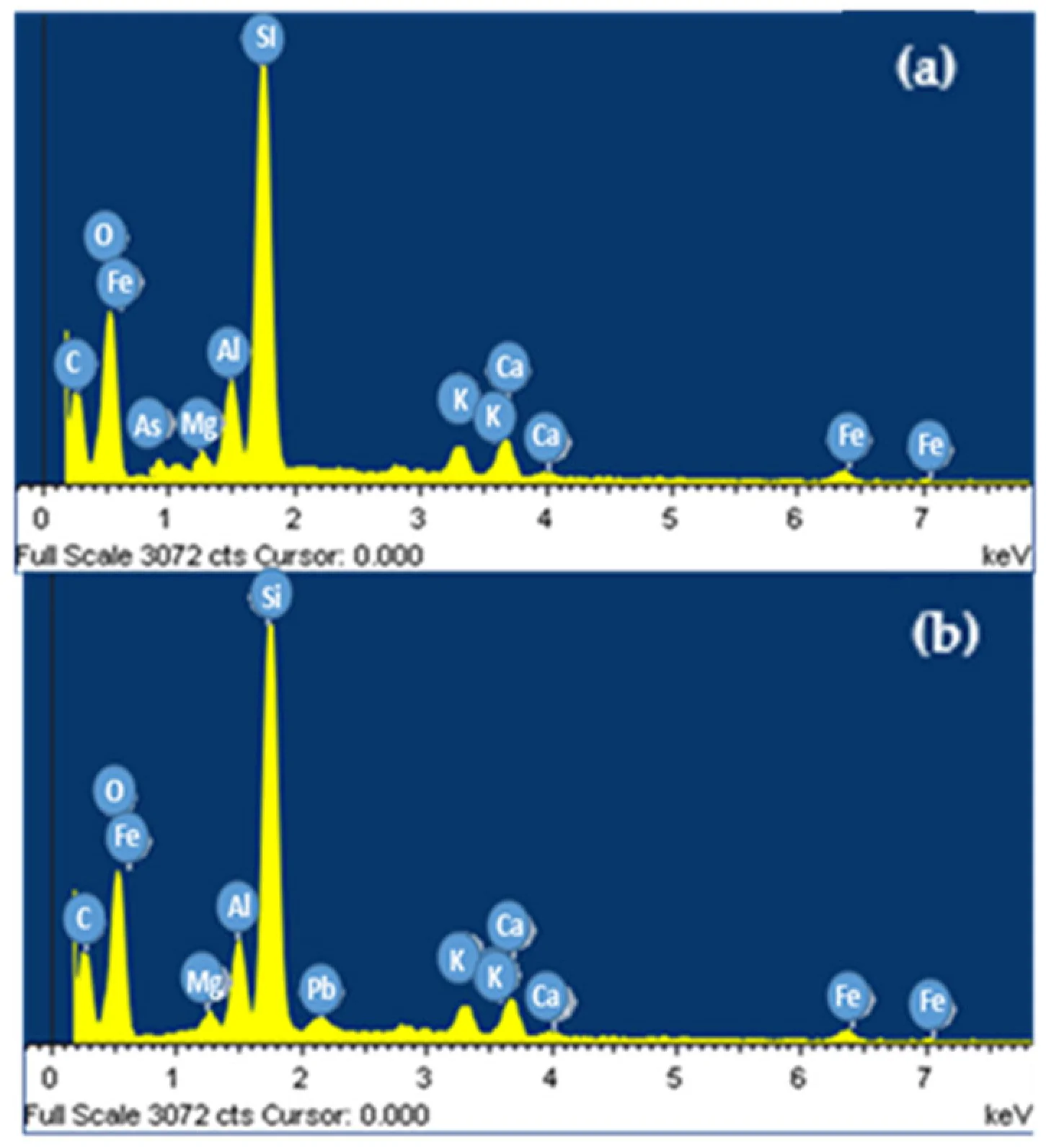
EDS spectra of zeolite showing the retention of (**a**) arsenic and (**b**) lead in its structure.

Finally, XRD was performed on the zeolite to identify the presence of any compounds containing toxic elements. New phases containing these elements were also observed; however, the zeolite signals overlapped with those of arsenate and lead oxide, making it difficult to detect both. Figure 7 shows the patterns of both the clean zeolite and the zeolite used in the packed column. The EDS spectra of the zeolite show the characteristic elements of this compound, but also exhibit peaks for arsenic (Figure 8a) and lead (Figure 8b). The arsenic response was less intense, confirming the low retention of these toxic elements. The decrease in adsorption could primarily be due to the presence of atmospheric carbon dioxide (CO_2_), which readily carbonates the tetrahedral centers [41]. Zeolite is known for its ability to retain heavy metals; however, nanotubes have demonstrated greater retention efficiency.

### 3.2. Adsorption Capacity of As and Pb

#### 3.2.1. Effects of Time on As and Pb Adsorption

**Figure 9:**
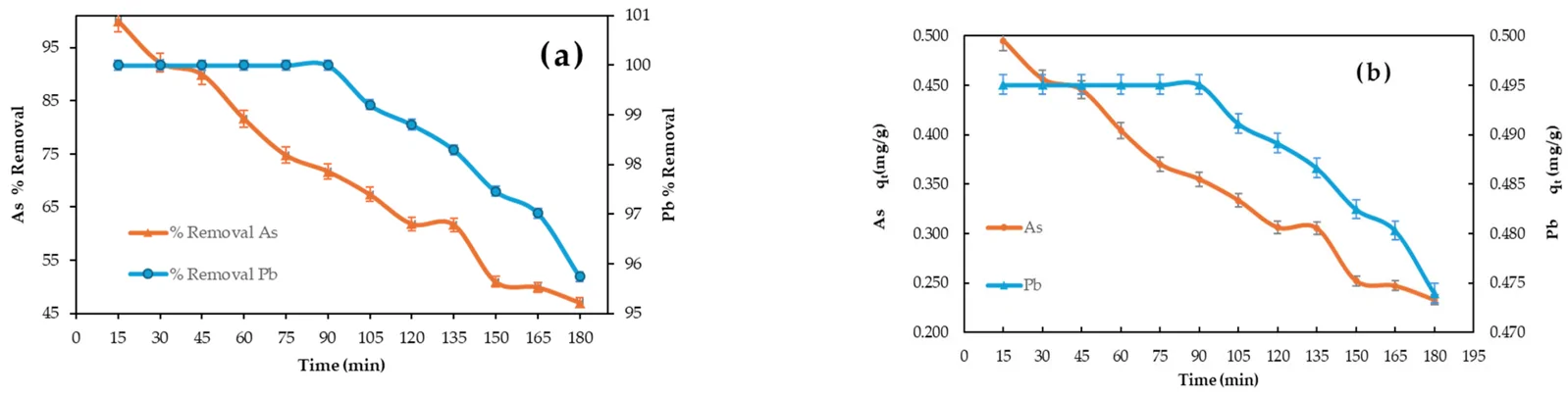
Effect of contact time on adsorption capacity and removal efficiency. (**a**) Removal (%) of As and Pb. (**b**) Adsorption capacity of As and Pb.

Contact time is a crucial parameter in adsorption processes. However, as the adsorbate passes through the packed-bed system, there is insufficient time to reach equilibrium with the adjacent adsorbent particles; therefore, the system must be designed to achieve rapid adsorption. The filtration time for 100 mL of solution under continuous flow condition was 180 min. The effect of contact time on the adsorption of As and Pb by the packed materials in the column is shown in Figure 9. The removal efficiency (%) for both metals is presented in Figure 9a. For As, the removal efficiency was 100% during the first 15 min and gradually decreased, reaching 47.04% at the end of the filtration process. This removal efficiency was higher than that reported by Payne et al. [41] using clinoptilolite in 48 h. For Pb, removal began at 100% and decreased slightly to 95.74% at the end of the filtration process. The adsorption capacity decreased over time, and both elements exhibited a similar trend (Figure 9b). For As, the capacity decreased to 0.232 mg/g, whereas for Pb it decreased only to 0.473 mg/g. The removal efficiency and adsorption capacity values are shown in Table 1.

The initial rapid stage was attributed to a process occurring at the particle surface, driven by the abundance of available adsorption sites. The slow stage, on the other hand, was attributed to anion exchange within the intermediate layer, which is expected to proceed more slowly than surface adsorption [42]. Metal ions gradually diffused into the adsorbent matrix until the active sites became saturated. Equilibrium was subsequently reached, resulting in a decrease in adsorption capacity [43,44,45]. Lead (Pb) exhibited higher adsorption efficiency than arsenic (As). As adsorption can be affected by the presence of phosphates, silicates, and organic matter, which may significantly influence arsenate adsorption because these anions compete with As for available adsorption sites [46].

#### 3.2.2. Dynamic Adsorption Experimental

**Figure 10:**
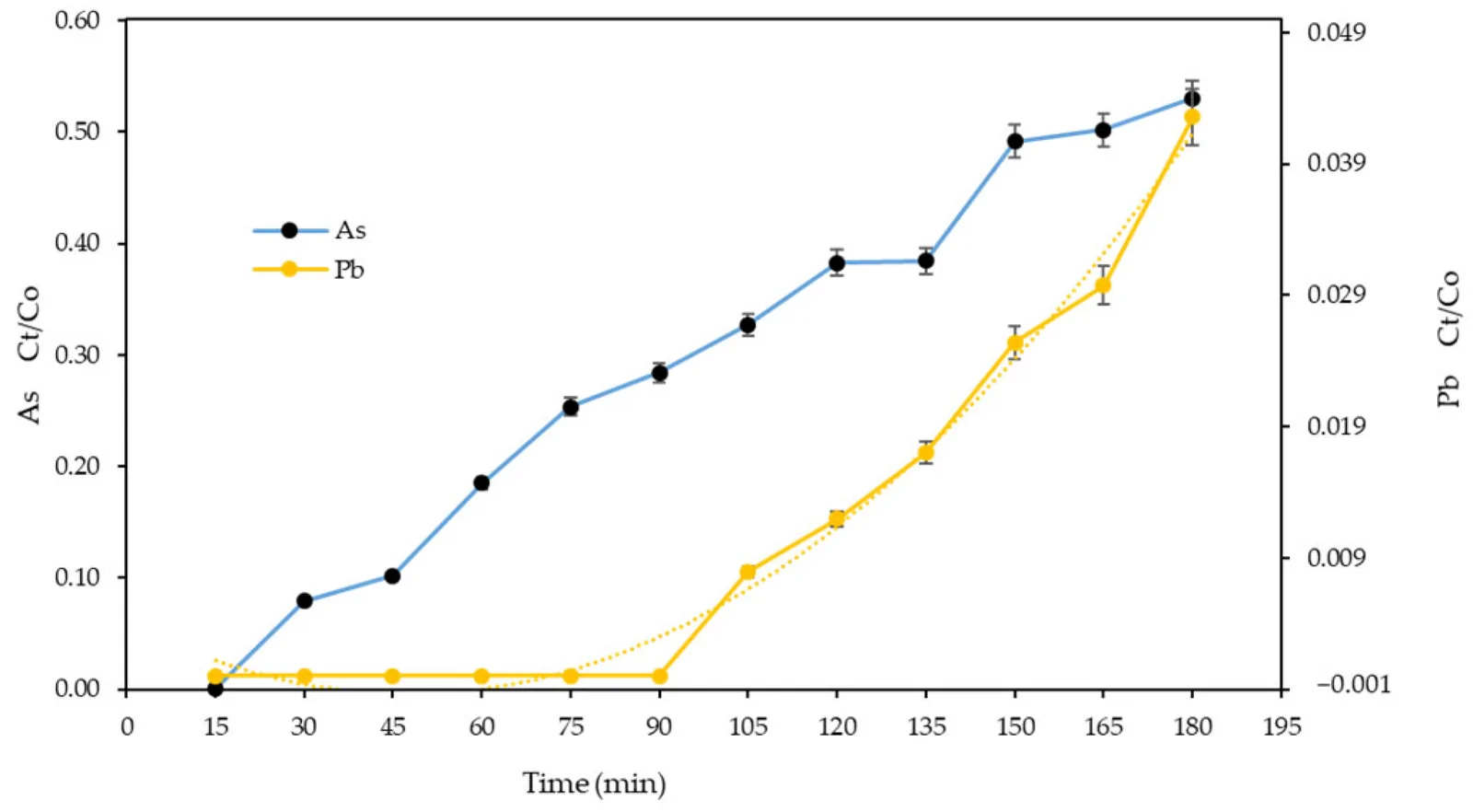
Breakthrough curves of arsenic and lead in the fixed-bed column.

The breakthrough curve describes the relationship between the outlet concentration and operating time of the fixed-bed column. The performance of a fixed-bed column is determined by several parameters, including the flow rate (Q); the bed height, which defines the mass transfer zone; and the initial adsorbate concentration. For As and Pb, the initial concentration was 50 mg/L, the total adsorbent mass was 10.1 g, and the bed height was 5 cm. For As, the breakthrough curve was less steep, whereas for Pb, more evident breakthrough behavior was observed (Figure 10). As filtration proceeded, the adsorption process gradually slowed, resulting in an increase in outlet concentration due to the saturation of available adsorption sites. The curves did not form the typical S shape because the saturation point was not reached, resulting in shortened mass transfer, where axial dispersion is considered negligible at the operating concentration [47]. A characteristic S-shaped breakthrough curve indicates efficient adsorption performance; a more pronounced S shape is associated with better column efficiency [48]. As the influent concentration increases, the available adsorption sites become occupied more rapidly, requiring a greater bed height. However, the flow rate must also be considered, since higher velocities reduce the contact time between the adsorbent and adsorbate [49,50].

#### 3.2.3. Kinetic Modeling

**Figure 11:**
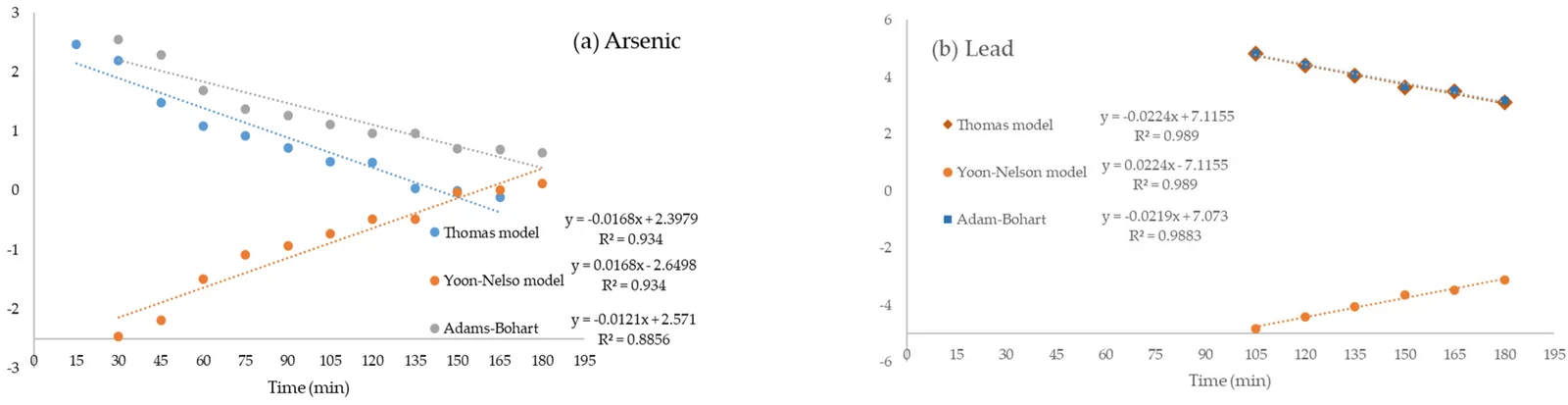
Thomas, Yoon–Nelson, and Adams–Bohart models used to fit the experimental adsorption data, predict breakthrough curves, and evaluate the efficiency and practical applicability of the adsorption columns. (**a**) Arsenic and (**b**) lead.

The experimental data obtained were analyzed using the Thomas, Yoon–Nelson, and Adams–Bohart models. The correlation coefficient values obtained from these models, which describe the breakthrough curve behavior, are shown in Table 2 and Figure 11 for As and lead.

The Thomas model was used to estimate the maximum adsorption capacity (q_0_) (0.2577 mg/g of As; 0.4240 mg/g of Pb) and the kinetic constant k_Th_ (0.000336 for As; 0.000448 for Pb). For arsenic, the Thomas model showed a satisfactory fit, with an R^2^ value of 0.934, suggesting that the adsorption process was not limited by chemical reaction kinetics but rather controlled by mass transfer mechanisms at the solid–liquid interface and is consistent with a second-order adsorption kinetic assumption [51]. The maximum adsorption capacity was low compared to some modified adsorbents (Table 3); this could be attributed to the short residence time (flow rate of 0.33 mL/min) and the initial concentration. The R^2^ value obtained for lead was 0.989, indicating that the breakthrough curve exhibited behavior characteristic of a system with high removal capacity during the first phase of the test. During the first 90 min, no lead concentration was detected, indicating that the adsorbents’ active sites had not yet reached saturation. After 105 min, a gradual increase in the effluent concentration was observed, reaching a maximum of 2.129 mg/L at 180 min (C_t_/C_0_ of 0.0426). This indicates that the adsorbent bed retained a high adsorption capacity, as the final effluent concentration represented only 4.26% of the influent concentration. Therefore, the column did not reach breakthrough, and the adsorbent bed remained unsaturated under the evaluated operating conditions.

The Yoon–Nelson model for arsenic described the intermediate region of the breakthrough curve, yielding a breakthrough time at 50% saturation of 157.8 min, which was consistent with the experimental data showing a C_t_/C_0_ ratio of 0.50 at 150 min. An R^2^ of 0.934 was obtained. This high degree of agreement can be attributed to the model assumption that the probability of adsorption decreases proportionally with the probability of adsorbate breakthrough, without requiring detailed information about the physical characteristics of the bed. The model assumes that the adsorption of the target element is directly related to the probability of adsorbate retention and breakthrough [51]. The adsorption process described by this model is commonly associated with mass transfer and diffusion mechanisms within the porous structure. The Yoon–Nelson model has been widely applied to describe breakthrough curves in fixed-bed adsorption systems, particularly when granular activated carbon is used as an adsorbent [52]. The adsorption mechanism may involve ion exchange and electrostatic interactions between metal ions and functional groups within the porous structures, along with interactions associated with van der Waals forces [53]. For lead, a k_YN_ of 0.0224, a breakthrough time (τ) of 317.65 min, and an R^2^ value of 0.989 were obtained. Although a high R^2^ value was achieved, a complete sigmoidal breakthrough curve was not observed. This indicates that a longer operating time than the estimated 50% breakthrough time would be required to reach complete breakthrough conditions.

The Adams–Bohart model allows an evaluation of the initial stage of the breakthrough curve, where adsorption is controlled by the available bed capacity and adsorbate concentration [51]. The R^2^ values obtained using the Adams–Bohart model were 0.886 for As and 0.988 for Pb. The estimated values of the kinetic constant (k_AB_) and the bed volumetric capacity (N_0_) are presented in Table 2. As expected, as the operating time and mass transfer increase, deviations between the model predictions and experimental data become more pronounced. For lead, the experimental data correspond mainly to the initial stage of the breakthrough curve; therefore, this model provided a more representative fit compared with the Thomas and Yoon–Nelson models. The Adams–Bohart model adequately describes the initial breakthrough region, whereas the Thomas model provides a better description of the intermediate region of the curve, and the Yoon–Nelson model is more suitable when data near 50% breakthrough are available.

The comparison of adsorbents for the removal of As and Pb is presented in Table 3. Differences in breakthrough times reported among the various studies are mainly attributed to factors such as adsorbent type, particle size, bed porosity, feed flow rate, initial adsorbate concentration, solution pH, and operating temperature. These variables directly influence mass transfer rates and adsorption capacities. The present study exhibited a similar trend, characterized by an initial period in which no contaminant was detected, followed by a gradual increase in effluent concentration as the availability of active adsorption sites decreased.

**Figure 12:**
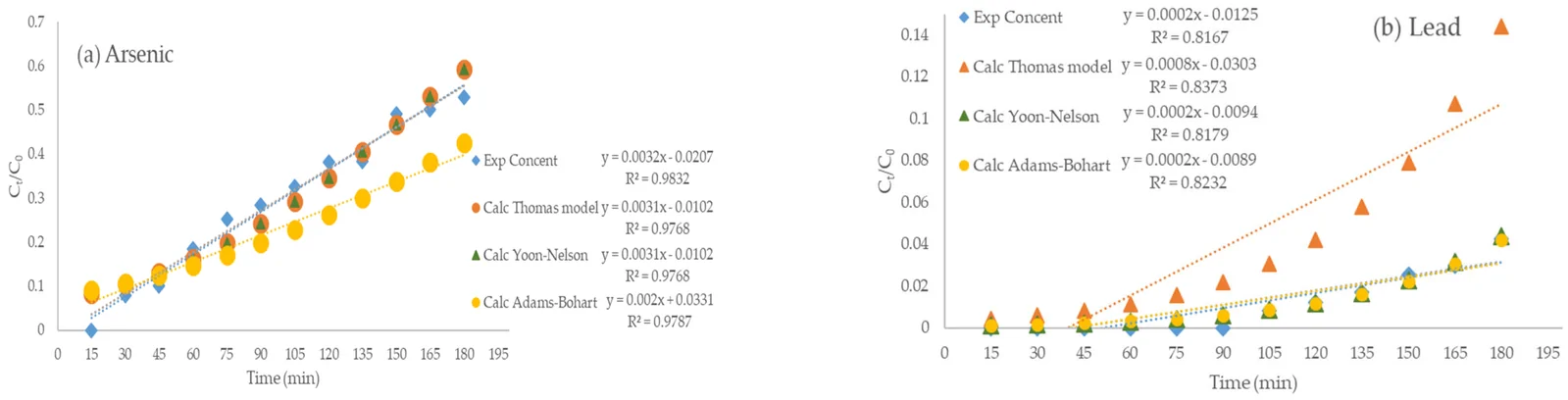
Breakthrough modeling using the Thomas, Yoon–Nelson, and Adams–Bohart models for (**a**) arsenic and (**b**) lead.

Figure 12 presents the breakthrough curve modeling and determination of the model constants. The dynamic adsorption behavior of the system was predicted using the Thomas, Yoon–Nelson, and Adams–Bohart models. Figure 12a,b show the experimental breakthrough curves (symbols) and fitted model curves (dashed lines) for arsenic and lead, respectively. The breakthrough curves predicted by the Thomas and Yoon–Nelson models showed significant differences compared with those obtained from the Adams–Bohart model. This difference is mainly attributed to the fact that the Adams–Bohart model describes the initial region of the breakthrough curve and tends to deviate as the system approaches saturation. In contrast, the Thomas and Yoon–Nelson models provide better descriptions of the intermediate region and the breakthrough time near 50% saturation, respectively.

## 4. Conclusions

In this work, the application of zeolite, activated carbon, and CVD-synthesized carbon nanotubes for the adsorption of arsenic (As) and lead (Pb) from water was investigated using a fixed-bed column. The materials were characterized by XRD and SEM before and after contact with synthetic As and Pb solutions. Carbon nanotubes and activated carbon showed adsorption of the target elements. Adsorption efficiencies for As ranged from 100% to 47%, whereas for Pb they ranged from 100% to 95%. The maximum adsorption capacity for both metals was 0.495 mg/g, decreasing to 0.2577 mg/g for As and 0.4240 mg/g for Pb. Based on the parameters obtained for arsenic, the fixed-bed column demonstrated functionality for As removal. For lead, the results indicate that the evaluated system has high potential for continuous treatment applications, since the effluent concentration remained well below the influent concentration ever after 180 min of operation. The operating time will be extended until bed saturation is reached (C_t_/C_0_ ≥ 0.90).

## Figures and Tables

**Table 1:** Removal efficiency (%) and adsorption capacity.

Time	As			Pb		
	[As] ppm	% Removal	q_t_ (mg/g)	[Pb] ppm	% Removal	q_t_ (mg/g)
15	0	100.000	0.495	0	100.000	0.495
30	3.923	92.154	0.456	0	100.000	0.495
45	5.038	89.924	0.445	0	100.000	0.495
60	9.191	81.618	0.404	0	100.000	0.495
75	12.627	74.746	0.370	0	100.000	0.495
90	14.16	71.680	0.354	0	100.000	0.495
105	16.324	67.352	0.333	0.398	99.204	0.491
120	19.089	61.822	0.306	0.598	98.804	0.489
135	19.166	61.668	0.305	0.851	98.298	0.486
150	24.533	50.934	0.252	1.270	97.460	0.482
165	25.047	49.906	0.247	1.488	97.024	0.480
180	26.480	47.040	0.232	2.129	95.742	0.473

**Table 2:** Model parameters and correlation coefficients for fixed-bed column models.

				Thomas			Yoon–Nelson			Adams–Bohart		
Heavy Metal	Q (mL/min)	H (cm)	C_i_ (mg/L)	q_0_ (mg/g)	k_TH_ (mL mg^−1^ min^−1^)	R^2^	k_YN_ (min^−1^)	τ (min)	R^2^	k_AB_ (L mg^−1^ min^−1^)	N_0_ (mg/L)	R^2^
As	0.33	5	50	0.2602	0.000336	0.934	0.0167	157.80	0.934	0.000242	7408.39	0.886
Pb				0.4240	0.000448	0.989	0.0224	317.65	0.989	0.000438	11281.83	0.988

**Table 3:** Comparison of adsorbents for the adsorption of As and Pb ions.

					Thomas		Yoon–Nelson		Adams–Bohart	
Heavy Metal		Q (mL/min)	H (cm)	C_i_ (mg/L)	k_TH_ (mL∙mg^−1^ min^−1^)	q_e_ (mg/g)	k_YN_ (min^−1^)	Τ (min)	k_AB_ (L∙mg^−1^ min^−1^)	N_0_ (mg/L)
Pb	Bioadsorbents [51]	10	9.5	20	2.36000		0.04700	89.40		
Pb	Biopolymeric aerogels [52]	1	5.0	20	0.00067	70.86	0.01300	1775.37	0.0005	7535.48
Pb	Iron oxide-coated zeolite [49]	11	6.0	60	0.72100	12.40			0.9980	3172.00
Pb	Composite alginate beads [53]	5	4.5	75	0.00015	136.40	0.03490	322.90		
Pb	Mg-Al LDH-doped activated carbon composites [54]	30		30	0.57900	37.50	0.36700	611.50		
As Pb	Carboxymethylated cellulose [55]	15	5.0	10		26.50 89.70				
As	Chitosan [56]	2	13.2	60	0.50200	43.30	0.03010	832.80	0.232	4020.30

## Data Availability

All data presented are original.
